# Coexistent Papillary Carcinoma of Thyroid and Hashimoto’s Thyroiditis – Diagnosis on Fine Needle Aspiration Cytology

**DOI:** 10.5812/ijem.7453

**Published:** 2013-07-01

**Authors:** Rumana Makhdoomi, Farhat Mustafa, Rais Malik, Salma Bhat, Khurshid Alam, Humaira Bashir, Nuzhat Samoon, Mohsin Rasool, Khalil Mohammed Baba

**Affiliations:** 1 Department of Pathology, Sher-i-Kashmir Institute of Medical Sciences, Srinagar-190 011, India; 2 Department of Surgery, Sher-i-Kashmir Institute of Medical Sciences, Srinagar-190 011, India

**Keywords:** Papillary Carcinoma Thyroid, Hashimoto’s Thyroiditis, Fine Needle Aspiration Cytology

## Abstract

Hashimoto’s thyroiditis is associated with an increased risk of developing papillary carcinoma of thyroid. We hereby report a case of Hashimoto’s thyroiditis with papillary carcinoma in a 45-year-old ear old female diagnosed on fine needle aspiration cytology (FNAC) which was later confirmed on histopathological examination .Such an occurrence, when both lesions are picked up on FNAC in a patient with no palpable thyroid nodule is rare. The case is presented here for its rarity.

## 1. Introduction

Hashimoto’s thyroiditis (HT), also called chronic lymphocytic or autoimmune thyroiditis, is part of the spectrum of autoimmune thyroid diseases (AITD) and is associated with various degrees of thyroid hypofunction (1). The relationship between Hashimoto’s thyroiditis and papillary carcinoma of thyroid was first proposed by Dailey et al in 1955 (2). Some studies have determined a clear association between the two diseases (3) whereas some studies have not found a clear association (1). Fine-needle aspiration e aspiration biopsy (FNAC) is recommended for patients with solitary ‘cold’ nodules, because of potentially increased risk of thyroid cancer (1). American Thyroid Association (ATA) recommends FNAC as the procedure of choice for evaluating thyroid nodules and selecting candidates for surgery (4). Fine needle aspiration cytology is useful for diagnosing PTC in patients with HT-associated thyroid lesions with a sensitivity of more than 90% (5). Fine needle aspiration cytology was useful in our patient for the diagnosis of coexistent Hashimoto’s thyroiditis and papillary carcinoma of thyroid although there was diffuse enlargement of thyroid without any evidence of a solitary ‘cold’ nodule.

## 2. Case Report

A 45-year-old female came to the surgical OPD with diffuse enlargement of thyroid gland which was more on left side than right. Patient had noticed this swelling for the last two months and there was a history of difficulty in swallowing since one month and an increase in the swelling over the last two weeks. Patient gave a history of hypothyroidism for which she was taking 100 microgram of thyroxine for the last two years. The patient was on a regular endocrinological follow-up. There was no history of pain, loss of weight, palpitations, giddiness, hoarseness of voice or intolerance to heat. There was no history of irradiation to heat and neck region.

On examination, there was diffuse enlargement of thyroid. The gland was visible in normal positioning of the neck (WHO –Grade2 enlargement) measuring about 3.5 cms in its greatest diameter on right side and 3 cms on the left side, it was, firm, non-pulsatile and non-tender which moved with deglutination. No nodule or cystic area was palpable. Ultrasound neck showed diffuse enlargement of the thyroid gland with right lobe measuring 30 mm and left lobe measuring 28 mm.

The routine laboratory investigations were normal, including thyroid function tests. Her level of antithyroid peroxidase antibody (TPO) was 65 u/mL.

**Figure 1.**
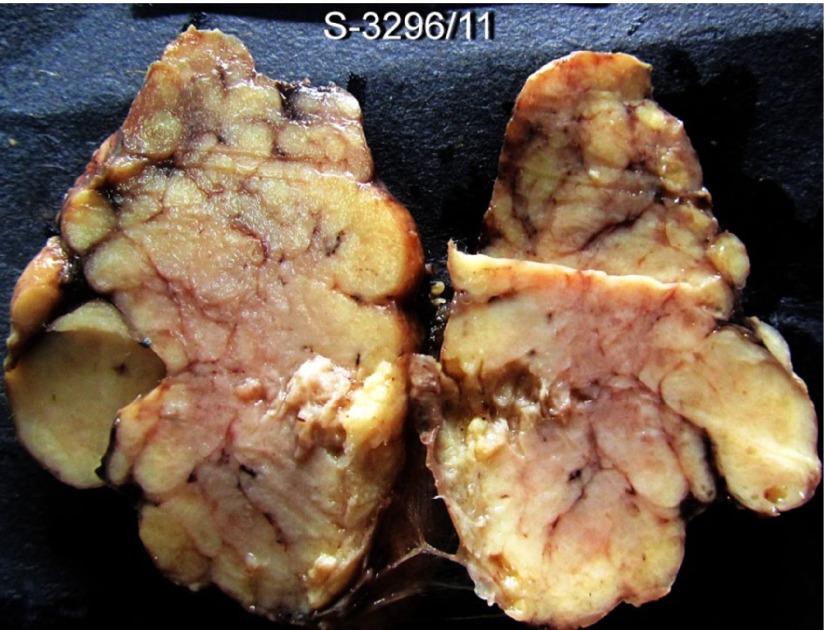
Fine-Needle Aspiration Smears Showing Dense Lymphocytic Infiltrate With Hurthle Cell Change (MGG 10X)

**Figure 2.**
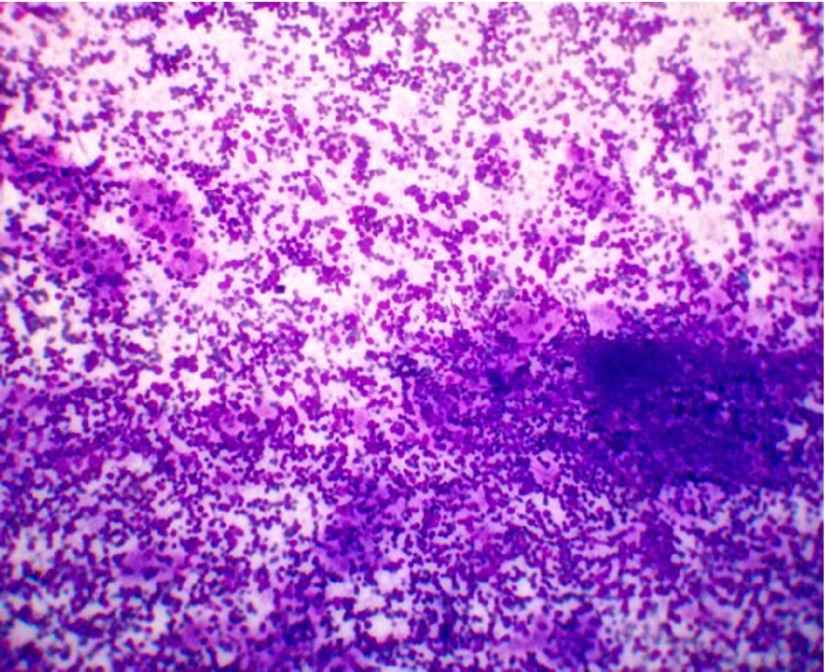
Fine-Needle Aspiration Smears Showing Dense Lymphocytic Infiltrate With Hurthle Cell Change (MGG 20X)

Fine-needle aspiration of the left lobe of thyroid was done because of the increase in the size of the gland on this side as reported by the patient leading possibly to the difficulty in swallowing. It was performed using a 22G needle. The smears showed hurthle cells in sheets with dense polymorphic lymphoid infiltrate comprising of mature lymphocytes, centrocytes, centroblasts , immunoblasts and tingible body macrophages (Figures 1, 2).

**Figure 3.**
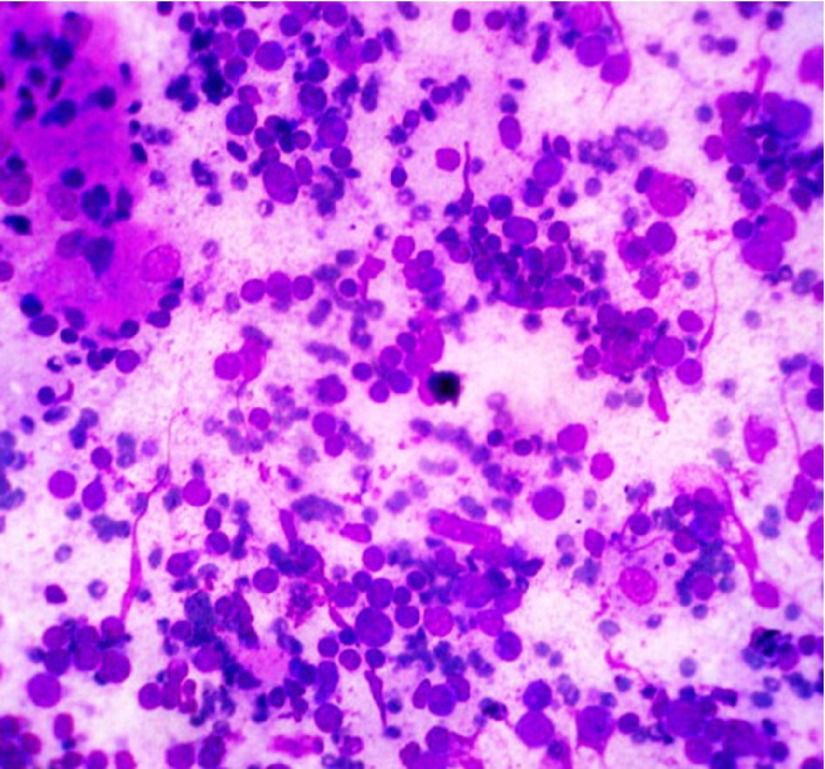
Photomicrograph Showing a Focus of Papillary Carcinoma With Hashimoto’s Thyroiditis (MGG 40X)

A few thyroid follicular cells were seen and there was a clump of cells with abundant cytoplasm, showing nuclear grooving and large hyperchromatic nuclei with prominent intranuclear cytoplasmic inclusions (Figure 3). An FNAC diagnosis of papillary carcinoma with Hashimoto’s thyroiditis was suggested, and patient underwent total thyroidectomy.

**Figure 4.**
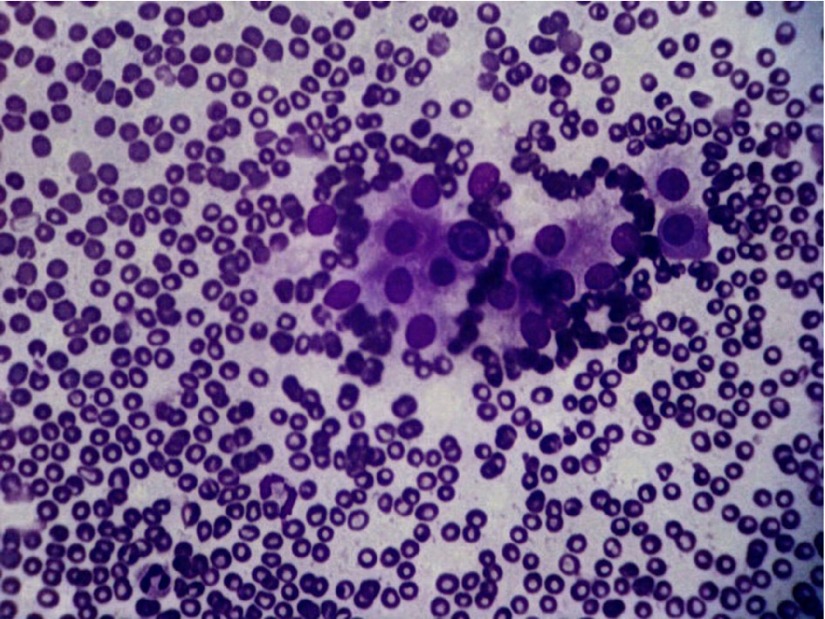
Gross Picture of Thyroid Showing Replacement of the Normal Architecture With Nodules of Varying Sizes Intraoperative findings: Both lobes of thyroid were enlarged, especially on the left side and the consistency was firm to hard.

Gross/histopathology: Total thyroidectomy specimen was grey white in colour, firm in consistency. Right lobe measured 5x3 cm and left lobe measured 5x4 cms with isthmus measuring 1 cm. External surface was rough and nodular. Cut section was grey white and entire thyroid was replaced by multiple nodules measuring a size from 0.5 cm to 1x1 cm (Figure 4). No normal thyroid parenchyma was identified. The capsule of the gland appeared intact. Random sections were taken from both lobes. Most sections showed polymorphic lymphocytes in varying stages of maturation arranged in follicles and replacing the normal thyroid tissue. Hurthle cells identified by abundant cytoplasm were seen in clusters and sheets. One of the sections from left lobe of thyroid showed two small foci of papillary carcinoma characterized by clear cells arranged in a fibrovascular core and showing a papillary architecture surrounded by large lymphoid follicles, some showing prominent germinal centres. Thus, the final diagnosis was papillary carcinoma of thyroid with Hashimoto’s thyroiditis. 

## 3. Discussion

Hashimoto’s thyroiditis (HT) is the most common inflammatory thyroid disease as well as the most common cause of hypothyroidism in the United States, as it affects 22 per 1,00,000 individuals (5). Papillary carcinoma of thyroid is the most prevalent form of cancer thyroid representing 70-80% of all diagnosed thyroid cancers (6) and is more frequent in women as has been reported in some studies (1).

Many studies have been undertaken to find out the relationship between Hashimoto’s thyroiditis and papillary carcinoma of thyroid, (2, 3, 6) and some studies (3, 6) have found a clear-cut relationship between these two entities, and some studies have shown that women with HT have a 30% increased risk of having PTC as compared with women without HT (6).

The incidence of neoplasia in Hashimoto’s thyroiditis diagnosis by fine needle aspiration cytology is 4% (7). Papillary carcinoma of thyroid and Hashimoto’s thyroiditis do not commonly co-exist on cytological examination. Chen et al. (8) have described co-existence of papillary carcinoma of thyroid and Hashimoto’s thyroiditis in a 56-year-old female, but the patient had bilateral thyroid nodules and in their patient, the diagnosis of Hashimoto’s thyroiditis and papillary carcinoma of thyroid was made on ultrasound guided FNAC whereas in our patient, the diagnosis was based on random FNAC of the left lobe of the patient.

Patil P et al. (9) also reports two cases of papillary carcinoma of thyroid co-existing with hashimoto’s thyroiditis in a 22-year-old lady and 57-year-old lady. Both patients had nodules within the thyroid detected on ultrasonography, which represented on histopathology as foci of papillary carcinoma of thyroid and one of his cases showed specks of calcification. Both his cases were euthyroid whereas the case reported by Chen had features of hypothyroidism (8). Both cases reported in the literature are different from our case as our case did not have a palpable thyroid nodule, neither was a lesion detected on Ultrasonography. In our case the only indication for FNAC was an increase in the size of the gland as reported by the patient and a firm gland noted during the examination. On the histopathological examination we found two foci of papillary carcinoma in a gland affected predominantly by Hashimoto’s thyroiditis. Thus, although presence of a nodule in a patient of Hashimoto’s thyroiditis is a clear indication for FNAC, (1) we recommend that a sudden increase in the size of the gland and a firmness on palpation should also be taken as an indication for FNAC,as the gland may harbor a focus of papillary carcinoma. FNAC may thus help to pick even a papillary microcarcinoma

The salient cytological features of Hashimoto’s thyroiditis include a high cellularity, multiple Hurthle cell nests and lymphoid infiltrate (10). Cytological features of papillary carcinoma like papillary clusters and presence of intranuclear cytoplasmic inclusions and presence of nuclear grooves are a hall mark of papillary carcinoma (10). Psammoma bodies and thick colloid may also be present. In our case, though the features of Hashimoto’s thyroiditis were prominent, there were two isolated foci of cells showing features of papillary carcinoma. Though features like Psammoma bodies and thick colloid were absent, but we made the diagnosis of co-existent papillary carcinoma of thyroid with Hashimoto’s thyroiditis, which was confirmed on histopathological examination showing two isolated tiny foci (< 1 cm) of papillary carcinoma. Lymphocytic infiltration on cytological examination can occur in patients with papillary carcinoma (11) but the characteristic Hurthle cell sheets will not be seen in those cases in our case too, it was predominant Hashimoto’s thyroiditis, which had two tiny foci of papillary carcinoma. Thus papillary carcinoma thyroid and Hashimoto’s thyroiditis, can co-exist without producing a lesion in the thyroid which is palpable on examination or detectable on ultrasonography. Though ret. Proto-oncogenens activation has been suggested as a possible mechanism in Hashimoto’s thyroiditis, leading to the development of papillary thyroid carcinoma (12). Hashimoto’s thyroiditis is often associated with thyroid lymphomas and may occur with papillary carcinomas (13).Thyroid lymphomas will on FNAC have a predominant monomorphic lymphoid population

There is a need to be cautious if any features of papillary carcinoma are seen on FNAC smears and a thorough grossing of the thyroid specimens is recommended especially in patients who have Hashimoto’s thyroiditis, because as was seen in our case, there may be foci of microcarcinoma which may be missed if ample sections are not taken and careful grossing of the specimens is not done. 

## 4. Conclusions

Papillary carcinoma thyroid and Hashimoto’s thyroiditis can co-exist without producing a palpable thyroid nodule, and Papillary carcinoma may thus be present as non-visible foci. There is a need to be cautious if any features of papillary carcinoma are seen on FNAC smears, and a thorough grossing of the thyroid specimens is recommended especially in patients who have Hashimoto’s thyroiditis. 
